# Marchiafava-Bignami and Alcohol Related Acute Polyneuropathy: The Cooccurrence of Two Rare Entities

**DOI:** 10.1155/2016/5848572

**Published:** 2016-09-07

**Authors:** Samine Boloursaz, Sirous Nekooei, Farrokh Seilanian Toosi, Hossein Rezaei-Dalouei, Behrooz Davachi, Sahar Kazemi, Bita Abbasi

**Affiliations:** Ghaem Hospital, Faculty of Medicine, Mashhad University of Medical Sciences, Mashhad, Iran

## Abstract

*Objectives*. The aim of this article is to represent the first reported case with cooccurrence of two rare alcohol related complications.* Case Report*. We report a 38-year-old man with chronic alcoholism who presented with both cranial and peripheral nerve palsy. On MRI examination characteristic findings of Marchiafava-Bignami disease were recognized.* Discussion*. Marchiafava-Bignami disease (MBD) is a rare complication of long-term, heavy alcohol abuse that has characteristic MRI findings. Acute alcohol related polyneuropathy (AARP) is another rare and not-well-understood complication of chronic alcohol abuse. We could not find any previous report of the cooccurrence of these two complications in the literature.

## 1. Introduction

Marchiafava-Bignami disease (MBD) is a rare complication of long-term, heavy alcohol abuse that results in decreased level of consciousness, seizure, and death. MBD is classically characterized by acute edema and necrosis of corpus callosum and subsequent symmetric demyelination and atrophy of this structure. The exact etiology of the disease is still under investigation [1].

Acute alcohol related polyneuropathy (AARP) is another rare and not-well-understood complication of chronic alcohol abuse. Despite the chronic polyneuropathy that is contributed to the malnourished state, the exact etiology of acute polyneuropathy in alcoholic patients is not well known and it is considered a variety of Guillain-Barre syndrome. AARP has been reported in only a few case reports [2–4].

In this study we describe cooccurrence of these two rare entities.

## 2. Case Presentation

A 38-year-old with a 14-year history of heavy alcohol abuse (average of 150 gr daily use) presented to the emergency room with acute ascending weakness in the lower limbs. The symptoms had started in the last two weeks and had exacerbated since. The patient was bedridden at the time of presentation. The family reported exacerbated episodes of behavioral changes in the last two weeks. On further examination the patient revealed a 10-day history of loss of appetite and difficult swallowing.

Neurological evaluation revealed altered mental state, flaccid muscle tone, and areflexia in the lower limbs. Muscle strength was 2/5 in the right and 1/5 in the left lower limb. We also detected reduced muscle strength in the upper limbs (4/5 in the right and 4/5 in the left upper limbs). There was also sensory impairment in the upper extremities to the level of both wrists. The gag reflex was impaired.

On laboratory examination anemia and impaired hepatic function were detected. Serum level of creatine phosphokinase (CPK) was normal. Serum electrolytes were normal.

Serum titres of vitamins B1, B6, B12 and Lead were normal. CSF analysis showed slightly elevated CSF protein (0.55 g/L) and normal white blood cell count. CSF protein level did not rise any further in the second tap.

Electrophysiological examinations revealed evidence of acute denervation (fibrillation and positive sharp waves), a pattern consistent with acute axonal polyneuropathy.

**Figure 1:**
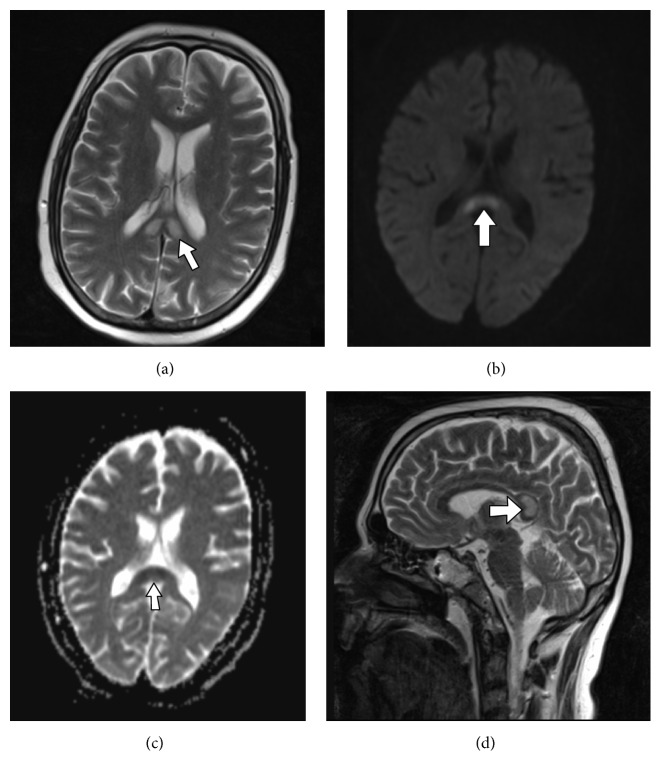
Axial T2-weighted brain MRI (a) shows increased signal in the splenium of corpus callosum (arrow in (a)). Diffusion weighted (DWI) (b) and ADC-map (c) images show restricted diffusion in the involved areas of corpus callosum (arrows in (a) and (b)) that is consistent with necrosis. Sagittal T2-weighted image (d) shows sparing of the periphery of corpus callosum.

The provisional clinical impression was alcohol related acute axonal polyneuropathy mimicking Guillain-Barre syndrome (GBS). As the cranial nerve involvement could not be explained, brain CT scan was performed and revealed a region of hypodensity in the splenium of corpus callosum. On the brain MRI an increased T2/FLAIR signal was seen in the splenium of corpus callosum (Figure 1(a)). The lesion showed restricted diffusion on diffusion weighted images (DWI) (Figures 1(b) and 1(c)). The periphery of the splenium was spared (Figure 1(d)). The findings were characteristic of Marchiafava-Bignami and explained the dysphagia and mood disorders.

Despite the normal level of serum vitamins, the patient was treated with vitamin B therapy and conservative management. After four weeks, he could walk, drink, and eat without difficulty and behavioral changes disappeared.

## 3. Discussion

MBD is a rare alcohol related disease, characterized by acute symmetrical demyelination and necrosis of the corpus callosum. The exact underlying etiology and pathophysiology still remain unclear. However some studies have suggested a number of risk factors including Lead toxicity, neoplasm, vitamin B complex deficiency, or osmotic disorders [1]. In our case we did not observe any history of neoplastic diseases and laboratory tests showed no Lead toxicity or vitamin B12 deficiency. Surprisingly, in the current study the diagnosis of acute alcohol related polyneuropathy was confirmed by EMG/NCV. In our literature search we could find just one study that reported cooccurrence of upper motor neuron disorders with Marchiafava-Bignami syndrome [5], but the cooccurrence of AARP and MBD has not been reported in the literature.

Although laboratory tests of our case did not show vitamin B deficiency, vitamin B therapy had a positive effect and clinical and imaging findings improved after four weeks of therapy. This suggests a common role of vitamin B deficiency in both AARP and MBD and may explain the cooccurrence of these two entities.
